# Iron-Catalyzed Coupling of Aryl Grignard Reagents with Alkyl Halides: A Competitive Hammett Study**

**DOI:** 10.1002/chem.201100467

**Published:** 2011-09-16

**Authors:** Anna Hedström, Ulla Bollmann, Jenny Bravidor, Per-Ola Norrby

**Affiliations:** [a] Department of Chemistry, University of GothenburgKemigården 4, 412-96, Gothenburg (Sweden), Fax: (+46)31-772-38-40 E-mail: pon@chem.gu.se; [b] Institute of Inorganic and Analytical Chemistry, Friedrich Schiller University JenaLessingstrasse 8, 07743, Jena (Germany)

**Keywords:** catalysis, coupling reactions, Hammett plots, iron, reaction mechanisms

The use of transition-metal catalysis to form new C–C bonds is an important tool in organic synthesis. Palladium- and nickel-catalyzed C–C bond-forming reactions have been extensively explored and are well understood.[1] The use of iron as the catalyst has gained much less attention, despite the innovative work of Kochi et al.[2] in the 1970s. Recently, several groups have turned their attention towards iron-catalyzed coupling reactions;[3] this growing interest in iron is due to its environmentally benign character, low cost, and non-toxicity. Furthermore, iron seems to allow all possible combinations of carbon hybridization in the coupling reaction.

We have recently published a mechanistic investigation into the iron-catalyzed coupling reaction between an aryl electrophile and an alkyl Grignard reagent.[4] A combination of reaction monitoring, a Hammett competition study, and DFT calculations indicated that the oxidation state of the catalytically active iron species is Fe^I^ and that the oxidative addition of the aryl halide is the rate-limiting step. The most important factor for achieving high conversion was slow addition of the Grignard reagent; fast addition caused precipitation of iron, presumably due to over-reduction.

Herein, we investigate the electronic effects on the nucleophile by use of a competitive Hammett study (Scheme 1). With the more weakly reducing aryl Grignards, it was found that the catalyst is stable in diethyl ether without additives.[5] Cyclohexyl bromide was added in aliquots to a mixture of *p-*substituted and unsubstituted phenyl magnesium bromide and consumed after each addition without adverse effects on the catalytic efficiency. Product formation was followed by GC, with samples taken before each addition of the electrophile.

**Scheme 1:**
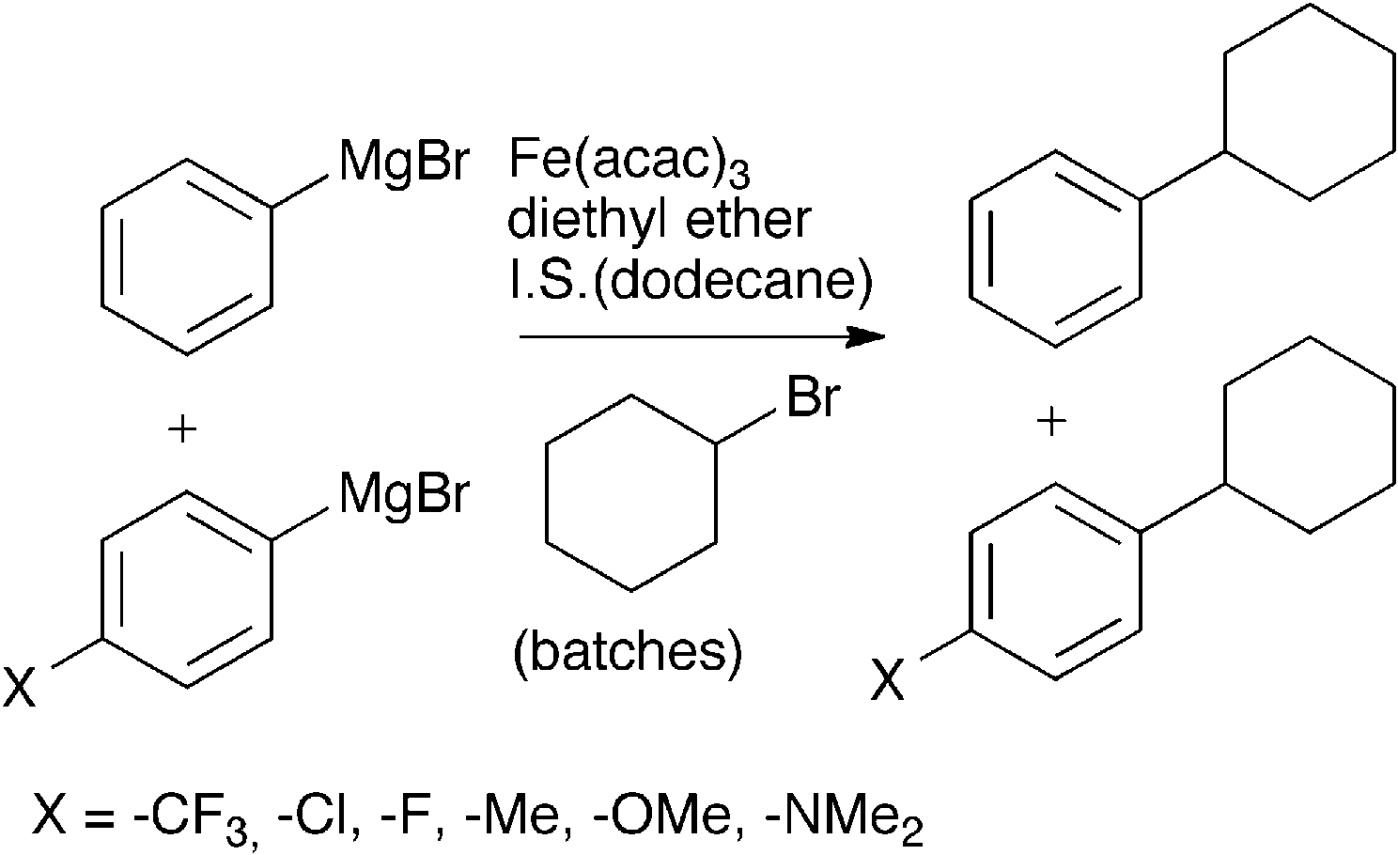
Competitive coupling of aryl Grignard reagents; I.S.=internal standard.

In the previous study employing strongly reducing alkyl Grignard reagents, catalyst deactivation could be visibly detected as a darkening of the solution, followed by precipitation. In this study, no deactivation was observed, despite the large excess of aryl Grignard present and the absence of stabilizing additives, demonstrating the lower reducing power of aryl Grignard reagents.

In analyzing the competition reaction data, we assumed that the kinetic dependence on all reagents and catalysts is the same for both substrates (X and H, for *p*-substituted and unsubstituted phenyl magnesium bromides) and that the reaction is first order in Grignard reagent. The relative rate (*k*_rel_=*k*_X_/*k*_H_) is then obtained as the slope of a plot of ln([X]_0_/[X]) against ln([H]_0_/[H]), that is, the initial and instantaneous concentrations of each Grignard reagent. Since these cannot be measured directly, they were calculated by comparing the instantaneous to the final product concentrations after addition of excess cyclohexyl bromide. Note that the analysis is insensitive to absolute concentration; only relative concentrations need to be well described. All plots gave straight lines with correlation coefficients *r*^2^>0.99 (Figure 1), indicating both that the assumptions we made were valid and that no significant side reactions occurred. Inspection of the trends indicates that some curvature can be detected in, for example, the case of the CF_3_-substituted Grignard, introducing a minor uncertainty in *k*_rel_ for this substituent, but it is clear from the plot that the possible deviation between the slopes in the initial and final phases of the reaction are too small to affect the conclusions drawn in this study.

**Figure 1:**
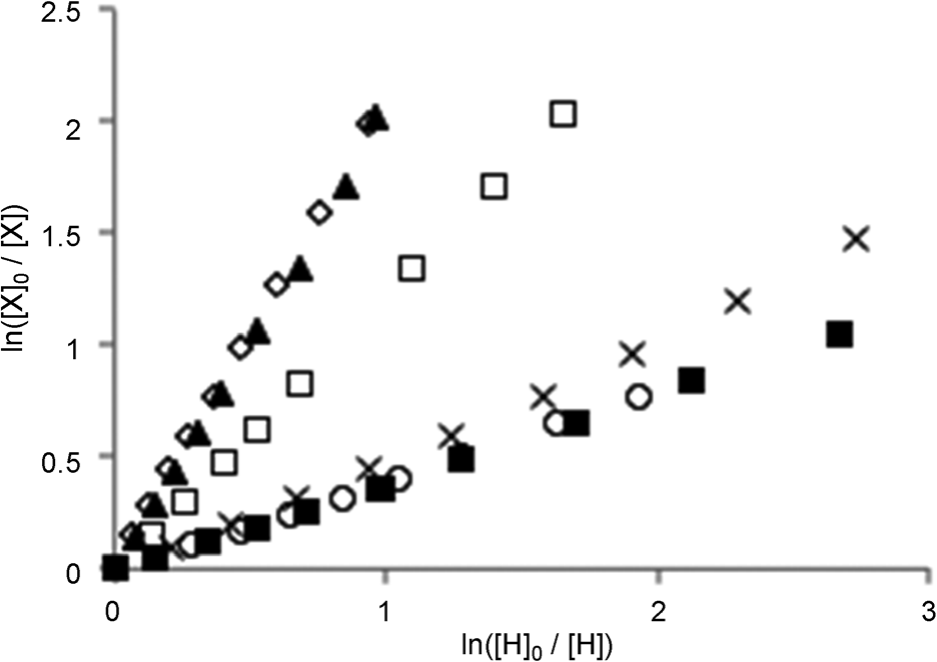
Plots of ln([X]_0_/[X])=*k*_rel_ln([H]_0_/[H]); ◊=OMe; □=Me; ▴=NMe_2_; ×=CF_3_; ▪=F; ○=Cl.

An alternative method of estimating the relative concentration of the Grignard reagent would be to analyze the amount of protonation product (benzene and substituted benzene) after workup. The protonation products are also produced in the initiation phase and therefore cannot be used directly as a measure of the Grignard concentration, but the concentrations should correlate after the initiation. We have therefore verified that the concentration of the protonation product at each point correlates with the concentration of the Grignard reagents estimated from product formation, providing a correlation coefficient of at least 98 %.

The relative rate, *k*_rel_, for each *p*-substituted phenyl Grignard reagent was fitted to the literature *σ* values[6] by using the Hammett expression log(*k*_X_/*k*_H_)=*ρσ*. The results are shown in Table 1 and depicted in Figure 2.

**Table 1:** Relative rates and *σ* values[6] for different *para* substituents

*para* Substituent	*σ*	*k*_rel_	log(*k*_rel_)
NMe_2_	−0.83	1.99	0.30
OMe	−0.27	2.13	0.33
Me	−0.17	1.22	0.09
F	0.06	0.39	−0.41
Cl	0.23	0.39	−0.41
CF_3_	0.54	0.51	−0.29

**Figure 2:**
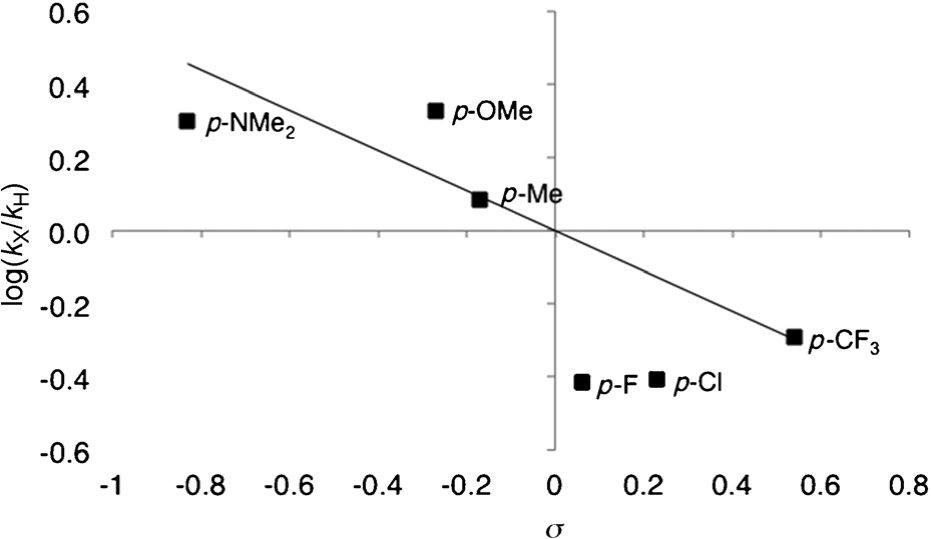
Hammett plot of log(*k*_rel_) versus *σ*.

As seen in Table 1, coupling is favored for Grignard reagents with electron-donating substituents (dimethylamine, methoxy, and methyl), whereas electron-withdrawing substituents (chloride, fluoride, and trifluoromethyl) inhibit the coupling reaction. This is in line with studies of transmetalation reactions in related palladium-catalyzed couplings,[7] and in agreement with the hypothesis of mechanistic similarity between the metals.[4]

Figure 2 shows that the fit to *σ* is less than perfect, and in particular that the halide substituents deviate. The latter observation is not uncommon in Hammett studies.[8] We tested several other *σ* scales (*σ*^+^, *σ*^−^, σ^**⋅**^, as well as Swain–Lupton parameters[9]), alone and in combination, but no statistically significant improvement could be obtained (as judged by using F tests). Fitting to the standard *σ* values gives *ρ*≈−0.5, varying slightly depending on whether the halides are included or not.

This Hammett study gives a weakly negative *ρ* value, which is in good agreement with previous studies of transmetalation reactions.[7] We note that the reductive elimination step is the same here as in our previous study; at this point the identity of the nucleophile and electrophile has been scrambled.[4] Since the Hammett study of the electrophile gave a positive *ρ* value, the reductive elimination cannot be the rate- or selectivity-determining step. In combination with the earlier computational study of reductive elimination from different iron species,[4] this gives strong support for an Fe^I^–Fe^III^ cycle. We note that a possible explanation for the unexpectedly low selectivity with the NMe_2_ substituent would be a change in the selectivity-determining step with this reagent; it is entirely possible that the high electron density here retards the reductive elimination enough to allow further transmetalation and thus negate the acceleration expected for oxidative addition in the presence of the NMe_2_ substituent.

Our previous study indicated that TM can occur either before or after OA with an sp^2^-hybridized electrophile, with very little difference in the two calculated energy barriers. In this case, OA to an sp^3^-hybridized electrophile is expected to be quite different and could potentially include either single-electron[5c, 10] or atom transfer, as recently indicated for Cu-catalyzed couplings.[11] A catalytic cycle of that type should produce an alkyl radical that could later couple. However, one good indicator of radical mechanisms is that all substituents are expected to provide stabilization and therefore should accelerate the reaction (i.e., most *σ*^**⋅**^ values are positive). In this case, however, we can exclude radical involvement in the coupling step, since we observe a decreased preference for nucleophiles containing electron-withdrawing groups.

The two possible TM steps shown in Scheme 2 are expected to show quite different behavior with respect to the electron density on the nucleophile, since one occurs at Fe^I^ and the other at Fe^III^. This could help rationalize the low correlation observed in Figure 2, as it is plausible that the preference displayed by the two iron species could change with electron density and therefore result in a change in the preferred reaction pathway. In particular, the deviation in selectivity for the CF_3_-substituted reactant could be a shift in preference from one OA pathway to the other. The local V shape in a Hammett plot is generally associated with a change in reaction pathway.

**Scheme 2:**
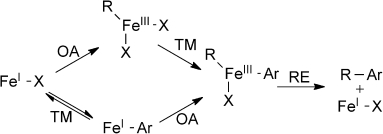
Proposed mechanistic pathways; oxidative addition (OA) followed by transmetalation (TM), or TM followed by OA. Reductive elimination (RE) gives the product and regenerates the iron catalyst.

In conclusion, this study supports a common mechanism for the two types of iron-catalyzed aryl–alkyl coupling reaction, based on an Fe^I^–Fe^III^ cycle. For both reactions, the relative ordering of the OA and TM steps is uncertain and path selection may be substrate dependent. Radical intermediates in the coupling step can be excluded, but we cannot draw any conclusions about the involvement of radicals in the oxidative addition step[10] as long as any radicals formed recombine with iron before the actual coupling occurs.

## Experimental Section

**General Procedure**: A round bottomed flask (50 mL) was sealed with a rubber septum, then evacuated and refilled with nitrogen four times. The flask was charged with diethyl ether (DEE) (30 mL), dodecane (225 μL, 1 mmol), phenyl magnesium bromide (400 μL, 1.2 mmol, 3 m in DEE), and *p*-substituted phenyl magnesium bromide (approx. 1.2 mmol). An aliquot (0.5 mL) was taken from the mixture, quenched with saturated NH_4_Cl (0.5 mL), filtered through a small silica plug, diluted with DEE and analyzed by GC (dodecane was used as the internal standard). A solution of Fe(acac)_3_ (18 mg, 0.05 mmol) and cyclohexyl bromide (0.2 mL, 0.1 mmol, 0.5 m in DEE) in DEE (5 mL) was added to the reaction vessel. After stirring for 5 min, an aliquot (0.5 mL) was collected and analyzed as described above. The procedure was repeated by adding cyclohexyl bromide (0.5 m in diethyl ether) in portions of 0.2 mL (a total of nine portions). Two extra portions of 1 or 2 equivalents of cyclohexyl bromide (122 μL, 1 mmol or 244 μL, 2 mmol) were added to react with any remaining Grignard reagent.
